# The Root Hair Specific SYP123 Regulates the Localization of Cell Wall Components and Contributes to Rizhobacterial Priming of Induced Systemic Resistance

**DOI:** 10.3389/fpls.2016.01081

**Published:** 2016-07-26

**Authors:** Cecilia Rodriguez-Furlán, Hernán Salinas-Grenet, Omar Sandoval, Camilo Recabarren, Paulina Arraño-Salinas, Sylvana Soto-Alvear, Ariel Orellana, Francisca Blanco-Herrera

**Affiliations:** ^1^Centro de Biotecnología Vegetal, Facultad de Ciencias Biológicas, Universidad Andrés BelloSantiago, Chile; ^2^FONDAP Center for Genome RegulationSantiago, Chile; ^3^Instituto de Investigaciones Agropecuarias-La PlatinaSantiago, Chile

**Keywords:** syntaxin, rhizobacteria, induced systemic resistance, systemic acquired resistance, cell wall, trafficking, PRP3, plant growth promoting rhizobacterium

## Abstract

Root hairs are important for nutrient and water uptake and are also critically involved the interaction with soil inhabiting microbiota. Root hairs are tubular-shaped outgrowths that emerge from trichoblasts. This polarized elongation is maintained and regulated by a robust mechanism involving the endomembrane secretory and endocytic system. Members of the syntaxin family of SNAREs (soluble *N*-ethylmaleimide-sensitive factor attachment protein receptor) in plants (SYP), have been implicated in regulation of the fusion of vesicles with the target membranes in both exocytic and endocytic pathways. One member of this family, SYP123, is expressed specifically in the root hairs and accumulated in the growing tip region. This study shows evidence of the SYP123 role in polarized trafficking using knockout insertional mutant plants. We were able to observe defects in the deposition of cell wall proline rich protein PRP3 and cell wall polysaccharides. In a complementary strategy, similar results were obtained using a plant expressing a dominant negative soluble version of SYP123 (SP2 fragment) lacking the transmembrane domain. The evidence presented indicates that SYP123 is also regulating PRP3 protein distribution by recycling by endocytosis. We also present evidence that indicates that SYP123 is necessary for the response of roots to plant growth promoting rhizobacterium (PGPR) in order to trigger trigger induced systemic response (ISR). Plants with a defective SYP123 function were unable to mount a systemic acquired resistance in response to bacterial pathogen infection and ISR upon interaction with rhizobacteria. These results indicated that SYP123 was involved in the polarized localization of protein and polysaccharides in growing root hairs and that this activity also contributed to the establishment of effective plant defense responses. Root hairs represent very plastic structures were many biotic and abiotic factors can affect the number, anatomy and physiology of root hairs. Here, we presented evidence that indicates that interactions with soil PGPR could be closely regulated by signaling involving secretory and/or endocytic trafficking at the root hair tip as a quick way to response to changing environmental conditions.

## Introduction

Soluble *N*-ethylmaleimide-sensitive factor attachment protein receptor (SNARE) molecules play an essential role in endomembrane fusion trafficking. Functional classification divides SNAREs into vesicle-associated SNAREs, termed v-SNAREs (members of the R-SNARE family), and target membrane-associated SNAREs or t-SNAREs (members of the Q-SNARE family), that interact to form a tetrameric bundle of coiled helices that draws the membrane surfaces together, facilitating fusion (Uemura et al., 2004; Lipka et al., 2007). There are three types of target membrane-associated Q-SNAREs that contribute to the formation of a SNARE complex: Qa-, Qb-, and Qc-SNAREs (Uemura et al., 2004). Qa-SNAREs are also termed syntaxins of plants (SYPs). The overall syntaxin structure consists of an N-terminal autoregulatory domain, a linker, the Qa-SNARE domain, and a transmembrane region (Lipka et al., 2007). In *Arabidopsis* there are five distinct SYP subfamilies. In the SYP1 family nine proteins (SYP111, SYP112, SYP121, SYP122, SYP123, SYP124, SYP125, SYP131, and SYP132) are localized at the plasma membrane (Uemura et al., 2004). Interestingly, SYP123 is exclusively expressed in trichoblasts, specialized epidermal root cells that, at the basal end, produce tubular-shaped root hairs (Enami et al., 2009). In these cells SYP123 was shown to be polarly localized to the tip of root hairs, which grow at rates of 1–2 μm/min (Galway et al., 1997). This elongation is extremely polarized and concentrated in a narrow tip growth zone (Shaw et al., 2000). Root hairs must deliver extensive amounts of cell wall material to the growing tip and constantly modify the preexisting cell wall, allowing the assembly and cross-linking of newly synthesized polysaccharides and proteins (Nielsen, 2008). Therefore, maintaining the root hair tip-focused growth rate requires the presence of an active secretory and endocytic system (Baluška et al., 2000; Ovečka et al., 2005; Park et al., 2011). Impairing the function of SYP123 inhibited root hair elongation, suggesting that SYP123 is closely related to the trafficking of cell surface materials during tip growth (Ichikawa et al., 2014). SYP123 has also been shown to cycle between the plasma membrane and brefeldin A (BFA)-sensitive endosomal compartments, indicating that is cycling in an endocytic recycling trafficking pathway. Nevertheless, there is no evidence regarding the potential cargoes delivered by the SYP123-regulated trafficking pathway in the growing root hair tip.

Root hairs play significant roles in nutrient and water uptake and increase the exploratory potential of the root system (Gilroy and Jones, 2000). These structures are also involved in the interactions between plants and soil-inhabiting microbiota, playing a critical role in root colonization by plant growth promoting rhizobacteria (PGPR; Prieto et al., 2011; Mercado-Blanco and Prieto, 2012). The PGPR drive post-embryonic root system architecture modifications by inhibiting primary root elongation and promoting lateral root and root hair formation (Zamioudis et al., 2013). Additionally, PGPR prime the aboveground plant parts to efficiently defend against a broad range of pathogens and insects (Conrath et al., 2006; Mendes et al., 2011), termed induced systemic resistance (ISR). Soil-borne *Pseudomonas* spp. are one of the most abundant PGPR capable of triggering the ISR signaling pathway (Mendes et al., 2011; Berendsen et al., 2012). Interestingly, observations of the *syp123* mutants in our growth chambers indicated that *Arabidopsis* plants were more susceptible to occasional pathogens. Little is known about the molecular mechanisms linking root hair colonization and ISR. Nevertheless, plasma membrane syntaxins have been previously related to plant pathogen defensive responses (Collins et al., 2003; Kalde et al., 2007; Kwon et al., 2008). SYP121/PEN1 is involved in plant extracellular immunity via exocytosis, participating in non-host penetration resistance against the powdery mildew *Blumeria graminis* f. sp. *hordei* and mediating focal secretion at *Blumeria graminis* f. sp. *hordei*–*Arabidopsis* interaction sites (Collins et al., 2003; Kwon et al., 2008). Alternatively, SYP132 in *Nicotiana benthamiana* contributes to bacterial pathogen resistance by mediating secretion of pathogenesis-related protein 1 (PR1; Kalde et al., 2007). Tobacco plants lacking SYP132 but not SYP121/PEN1 exhibit compromised bacterial resistance, suggesting that plants utilize distinct plasma membrane syntaxins against various pathogen types during immune responses (Kalde et al., 2007). Therefore, the role of SYP123 in priming the ISR signaling pathway by PGPR was analyzed.

The work presented here indicates that a deficiency in SYP123 function affected the arrangement of cell wall polysaccharides and protein localization at the tip of growing root hairs, and that SYP123 also aided in priming ISR upon PGPR exposure.

## Materials and Methods

### Plant Materials and Growth Conditions

Experiments were conducted with wild-type (WT) *Arabidopsis thaliana* (ecotypes Columbia, Col-0), *Arabidopsis prp3* knockout mutant (Larson et al., 2014) and *npr1.1* mutant (Cao et al., 1994) plants. The dominant negative (DN) of SYP123 was generated by cloning the Sp2 fragment, the CDS sequence lacking the coding region for the transmembrane domain, into an inducible expression system (Joubès et al., 2004). The cDNA of *Arabidopsis* roots was analyzed by PCR using the forward primer 5′-CACCATGAACGATCTTATCTCAAGCT-3′, and the reverse primer 5′-CTACCATTTCCTGTTGTTCCTCTGAAG-3′. The PCR DNA fragments were inserted into the pENTR/SD/D TOPO vector (Invitrogen, USA) and then subcloned into the plasmid pJCGLOX by GATEWAY technology (Joubès et al., 2004). All constructs were verified by sequencing. The plasmids were transferred into the *Agrobacterium tumefaciens* GV3101 strain and used for floral dip transformation of *Arabidopsis* Col-0 (Clough and Bent, 1998). Transgenic plants were obtained by kanamycin resistance and later transferred to soil for optimal seed production. The T3 *Arabidopsis* DN-SYP123 plants were induced by 10 μM of dexamethasone spray and, after 48 h, treated for 2 h at 37°C. Plants were analyzed 24 h after the duplicate induction. Induction was considered appropriate when GFP fluorescence in the nucleus disappeared.

The PRP3 gene was cloned using the forward primer 5′-CACCAGTTACTAATAAAACACCTTC-3′ and the reverse primer 5′-GAGCTCGTATTTGGGAGTGGCG-3′ and introduced into the pENTR/SD/D TOPO vector. Using GATEWAY technology, the entry vector was recombined with the pKGWFS7 vector creating a fusion in frame with GFP. Using the floral dip technique, *syp123* mutant plants were transformed with *Agrobacterium* carrying the PRP3-pKGWFS7 vector (Clough and Bent, 1998). Kanamycin-resistant plants were selected and T3 generation utilized in this paper.

An insertional mutant for SYP123 (At4G03330) was identified in Gabi-kat, NASC (Nottingham *Arabidopsis* Stock Center, N338418). Homozygous plants were selected in sulfadiazine 15 μg/mL. The presence of T-DNA was confirmed by PCR using the following primer pairs to amplify the WT allele: Forward 5′-CCATGAACGATCTTATCTCAAGCT-3′ and Reverse 5′-CTACCATTTCCTGTTGTTCCTCTGAAG-3′. The primers pairs applied to amplify the mutant allele were: Forward 5′-CCATGAACGATCTTATCTCAAGCT-3′ and Reverse 5′-ATATTGACCATCATACTCATTGC-3′.

PRP3-myc (donated by M. Tierney) were crossed with DN-SYP123 to generate transgenic plants (DN-SYP123xPRP3-myc).

Surface-sterilized seeds were sown in water for 48 h at 4°C, plated in Murashige and Skoog medium supplemented with 1% sucrose, and grown at 22°C under continuous light. Alternatively, 2-weeks-old seedlings were transferred to 60 ml pots containing a Sunshine number 3 potting soil mixture. The plants were grown in a growth chamber with a 16:8 h light:dark cycle, and irrigated twice a week.

### Immunolocalization

Protein detection in the cell wall was performed by fixing 4-days-old seedlings in 100% (v/v) methanol for 30 s at room temperature. Epitopes were blocked by incubating seedlings for 2 h with Tris-buffered saline [TBS; 20 mM Tris (pH of 8), 150 mM NaCl] plus 3% bovine serum albumin. Seedlings were incubated overnight with primary antibody 1:1000 anti-myc (Invitrogen, USA) in TBS and 0.5% bovine serum albumin. Washes were performed using phosphate buffered saline plus 1% Triton X-100. Then roots were incubated in secondary antibody anti-mouse-Alexa488 (Invitrogen, USA) diluted 1:1000 in TBS 0.5% BSA. Seedlings were washed using phosphate buffered saline plus 1% Triton X-100 and mounted on 50% glycerol for confocal imaging.

Pectin detection in the cell wall was performed by fixing the cells in 4% paraformaldehyde, 50 mM Pipes, 5 mM MgSO_4_, and 5 mM EGTA. Blocking was performed using TBS plus 5% fat free milk. Overnight incubation utilized primary antibody 1:100 Jim5, Jim7, Jim11, Jim14, LM1, LM2, CCRCM1, or CCRCM4 in TBS and 0.5% fat free milk. Then, roots were incubated in secondary antibody anti-rat Alexa 488 diluted 1:1000 in TBS and 0.5% fat free milk. Washes were performed using TBS plus 0.05% Triton X-100. Seedlings were mounted on 50% glycerol for confocal imaging.

### Bioassays

*Pseudomonas* infection assays were performed as described previously (Blanco-Herrera et al., 2015). *Pseudomonas syringae* pv. *tomato* DC3000 (*Pst* DC3000) and *Pst* avrRpm1 were grown at 28°C in King’s B liquid media and supplemented with 50 mg/ml rifampicin and 50 mg/ml kanamycin. Bacteria were washed and resuspended in 10 mM MgCl_2_, adjusted to optical density (OD_600_ = 0.001), and pressure infiltrated into three to four leaves per plant leaf using a needleless syringe. Leaf disks from six independent plants were combined, ground into 10 mM MgCl_2_, serial-diluted 1:10, and plated onto King’s B medium containing the appropriate antibiotics (rifampicin and kanamycin). Plates were incubated at 28°C for 3 days, then the colonies were counted.

To test for SAR, 4–5-weeks-old plants were pre-inoculated with *Pst* avrRpm1 (OD_600_ = 0.001) or mock (10 mM MgCl_2_) 24 h prior to infection and subsequently inoculated with *Pst* DC3000 (OD_600_ = 0.001) into three to four distal leaves per plant and four plants per genotype. Sampling was performed 3 days post-inoculation (dpi).

*Botrytis cinerea* (Chilean isolate) were cultured in 0.05% glucose, 0.03 M KH_2_PO_4_ (pH of 5), and incubated at 25°C. Conidia were suspended with a spore density of 1 × 10^5^/ml. Leaves of 4-weeks-old *Arabidopsis* soil-grown plants were detached and placed in Petri dishes with petioles embedded in Murashige and Skoog 0.7% agar. Two droplets of spore suspension (5 μL each) were placed on the surface of each leaf and incubated at 23°C in a 12-h photoperiod and lesion diameter was measured after 4 days.

Cultures of *Phytophthora infestans* were routinely grown on potato agar medium supplemented with 2% sucrose. Zoospores were produced by flooding 14-days-old cultures with dH_2_O, followed by incubation at 4°C for approximately 3 h. Rosette leaves of 4-weeks-old plants were inoculated with 10 μl droplets of zoospore suspensions. Concentrations ranging from 200,000 to 500,000 zoospores/mL were used for all experiments. Deionized water was the negative control in all relevant experiments. Inoculated plants were transferred to a phytochamber with a 16:8 h light:dark cycle at 20°C for 3 days. The quantification of the resistance percentage was done as Schlaeppi et al. (2010).

### Cultivation of Rhizobacteria and ISR Induction Treatments

Plant growth promoting rhizobacterium-induced ISR assays were performed as previously described by Zamioudis et al. (2013). Non-pathogenic fluorescent rhizobacteria *Pseudomonas* spp. isolated were cultured on King’s medium B agar plates supplemented with 50 mg/ml rifampicin at 28°C. Single bacterial colonies were collected in 10 mM MgSO_4_, washed twice by centrifugation (5 min at 5,000 × *g*), and suspended in the same buffer with an adjusted optical density (OD_600_ = 0.002). Droplets of bacterial suspension (240 μl) were dotted 5 cm from the root tip of 4-days-old seedlings of each genotype. Seven days after co-culture plants were collected for further analysis.

### Histochemical Staining

To monitor the cell death response, pathogen-infected leaves were stained with trypan blue [0.1% trypan blue water:glycerol:lactic acid (1:1:1)] and distained with water:glycerol:lactic acid (1:1:1) solution. Diaminobenzidine (DAB)-staining of H_2_O_2_ was performed as described (Thordal-Christensen et al., 1997). Stained leaves were stored in 50% glycerol solution and examined by light microscopy using a microscope equipped with a digital CCD camera. Callose depositions were visualized using aniline blue [0.01% in 150 mM KH_2_PO_4_ (pH of 9.5)] as described by Adam and Somerville (1996). Stained leaves were stored in 50% glycerol solution in the dark and subsequently examined by confocal microscopy (excitation 330–380 nm, emission 420 nm).

### Imaging

Bright-field microscopy was carried out using an Olympus microscope connected to a digital CCD camera. For the rest of the experiments utilized the Olympus Fluoview 1000 confocal microscope and a UPLSAPO 10x, UPLSAPO 20x, UPLSAPO 60xW. The enhanced green fluorescent protein (EGFP) and Alexa-488 fluorophores were observed using the 488 nm laser (excitation 488, nm-emission 500–530 nm). Meanwhile, FM4-64 and EGFP were excited by a 488 nm laser using spectral parameters recollecting the emission for EGFP at 500–530 nm and from FM4-64 at 575–625 nm. Image analyses were performed using ImageJ/Fiji software v1.47 (National Institutes of Health, USA^1^).

### Quantitative PCR

After the respective treatment, frozen plants were homogenized in liquid nitrogen. Total RNA was isolated using TRIzol^®^ (Invitrogen, USA) according to the manufacturer’s instructions. Residual DNA was removed with RNase-free DNase I (Invitrogen, USA). The cDNA was synthesized from each sample (1 μg of total RNA) with RevertAid First Strand cDNA Synthesis Kit (Thermo Fischer Scientific, USA) following the manufacturer’s recommendations. Quantitative RT-PCR was performed using the Fast Eva Green^®^ Master mix (Biotium, USA) in an ECO Real-Time PCR System (Illumina, Inc., USA). The conditions for amplifications were as follows: 95°C for 10 min and 40 cycles at 95°C for 10 s, 55 or 58°C for 15 s, and 72°C for 15 s. Data were analyzed in Eco Study v5.1 software (Illumina, Inc., USA). To evaluate the expression level of *PR1, PDF1.2*, and *MYC2* genes, *EF1-α* was the housekeeping gene. Relative accumulation level, with respect to *EF1-α*, was determined using the Pfaﬄ equation (Pfaﬄ, 2001).

The following primers were designed for gene-specific transcript amplification:

*PR1* (At2g14610) *F*: AACATGTGGGTTAGCGAGAA, *R*: TACACCTCACTTTGGCACAT; *PDF1.2* (At5g44420) *F*: GCACTGATTCTTGCATGCAT, *R*: TGTTCTCTTTGCTGCTTTCG; *MYC2* (At1g32640) *F*: CCCCACCGGTTTAATCGAAG, *R:* CGAGCGGTTGTACCAAACG*; SYP123* (At4g03330) *F:* TTGTTAGAGCCCTTTCGATT, *R*: ATAAAGCAATTACAGTAGCAA; *EF1-α* (At4g03330) *F*: TCACCCTTGGTGTCAAGCAGAT, *R*: CAGGGTTGTATCCGACCTTCTT.

## Results

### SYP123 Is Necessary for Protein and Polysaccharide Localization at the Root Hair Cell Wall

**FIGURE 1:**
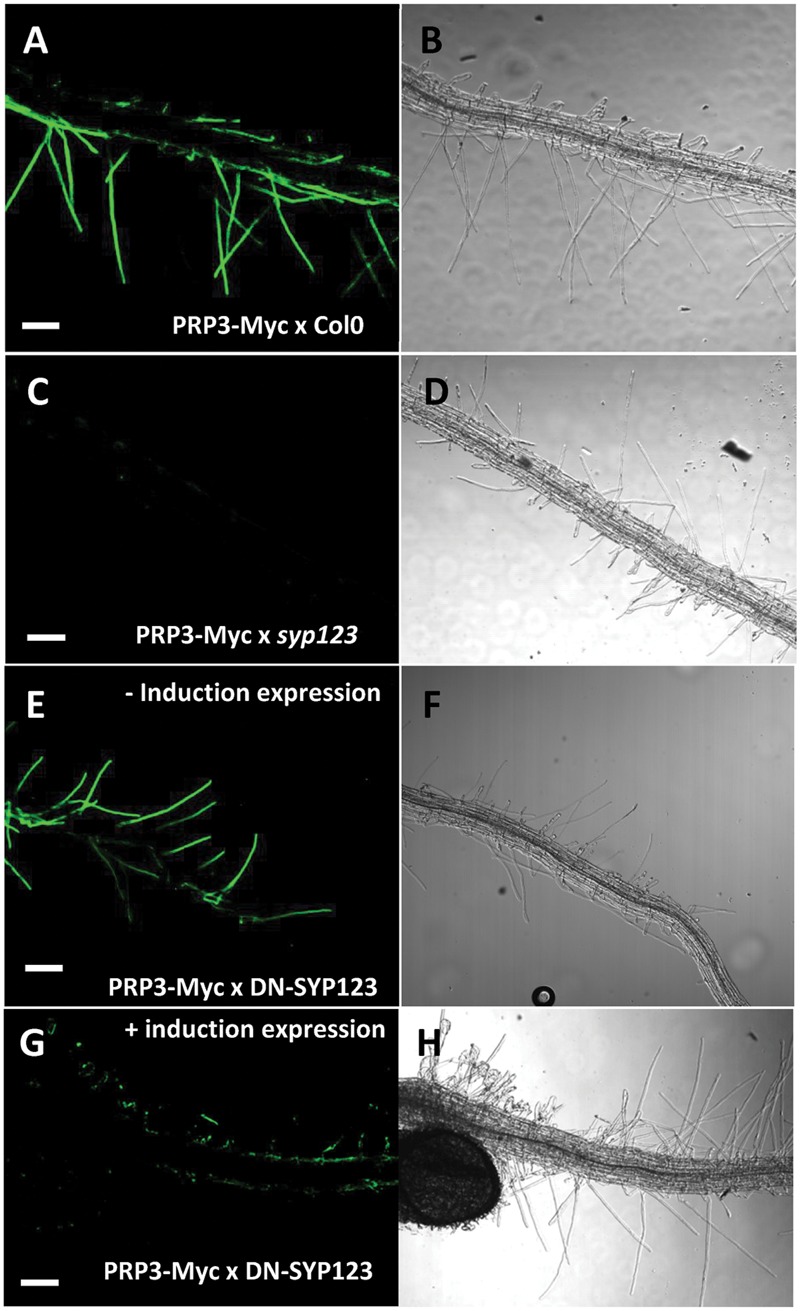
**SYP123 defective plants have altered PRP3 distribution at the root hair cell wall.** PRP3-Myc protein immunolocalization without permeabilization in roots of *Arabidopsis* seedlings in the Col-0 **(A,B)**, *syp123* **(C,D)**, the dominant negative (DN)-SYP123 with induction of the expression **(E,F)**, and the control without induction **(G,H)**. Immunolocalization was performed using Anti-Myc mouse monoclonal primary antibody and Alexa Fluor-488 conjugated goat anti-mouse IgG (green fluorescence left panel). Bright field images are displayed on the right panel. Scale bar = 50 μm.

**FIGURE 2:**
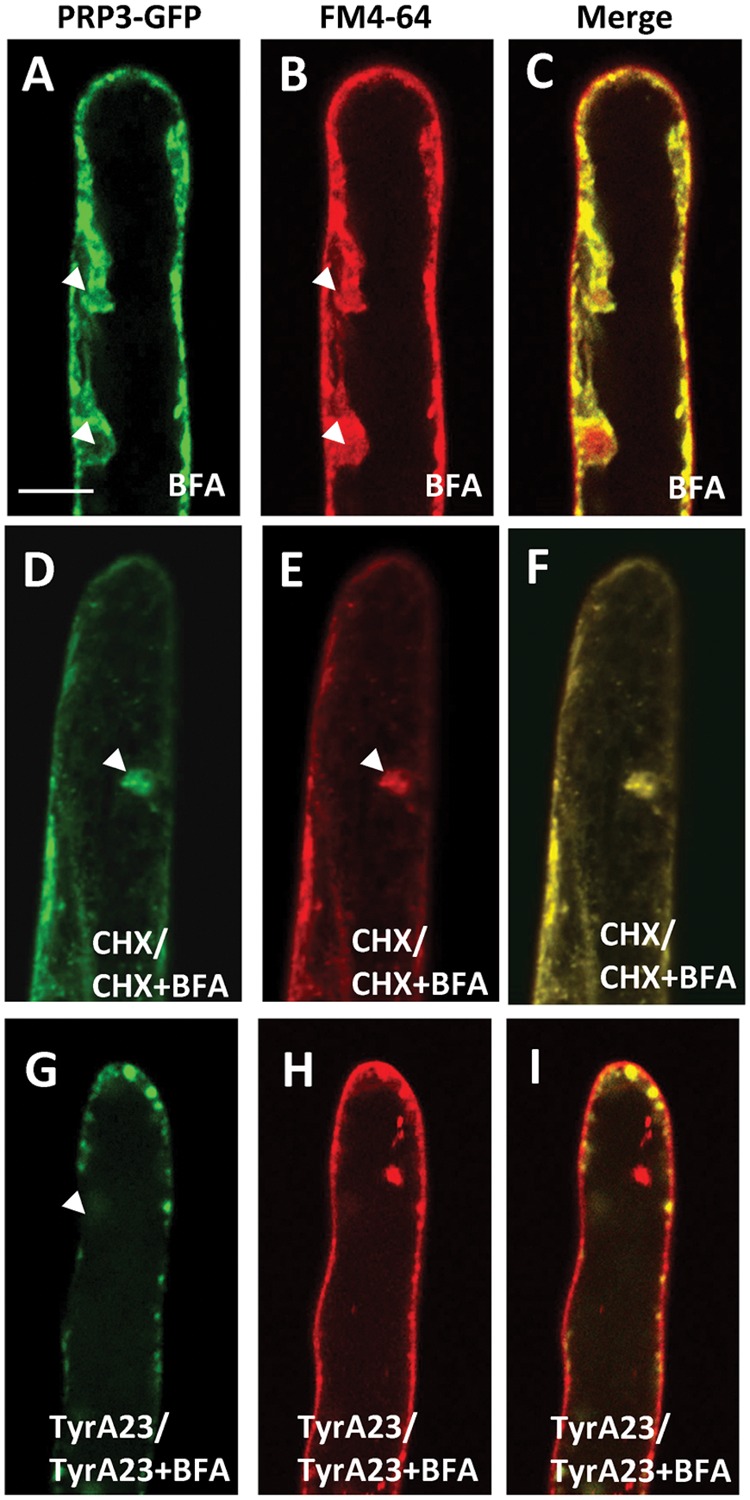
**Brefeldin A (BFA) treatment caused the internalization of PRP3-GFP.** PRP3-GFP **(A)** and FM4-64 **(B)** are present in BFA compartments, evidenced by the co-localization in the merged image **(C)**. Cycloheximide treatment inhibited protein synthesis but did not inhibit the internalization of PRP3-GFP **(D)** or FM4-64 **(E)** into BFA compartments as they still co-localize **(F)**. TyrA23 treatments efficiently inhibited BFA-induced intracellular accumulation of PRP3-GFP **(G)** and FM4-64 **(H)**. Merged image **(I)**. Scale bar = 10 μm.

The deposition and development of the cell wall is central to root hair tip growth and is highly coordinated with endomembrane trafficking in the cell. To test the involvement of SYP123 in cell wall deposition, the *syp123* knockout mutant plants were analyzed (Supplementary Figure S1). Additionally, a DN mutant (DN-SYP123) lacking the transmembrane domain (Sp2 fragment) was generated as previously described (Tyrrell et al., 2007) and controlled by an inducible system (Supplementary Figure S2). The localization of the root hair-specific structural cell wall protein PRP3 was analyzed in the mutant and DN backgrounds. Concurring with previous data (Albersheim et al., 2011), whole mount immunofluorescence detected PRP3-myc exclusively at the cell wall and concentrated at the tips of growing root hairs (**Figures 1A,B**). However, the PRP3-myc proteins in the *syp123* mutant were not detected in root hair tip cell walls (**Figures 1C,D**). In the induced-DN-SYP123 plant, PRP3-myc accumulation at the cell wall was significantly decreased in newly formed, growing root hair tips (**Figures 1G,H**) compared to uninduced-DN-SYP123 (**Figures 1E,F**). Since previous reports have found recycling SYP123 in Brefeldin A (BFA)-sensitive endosomes, the presence of PRP3-GFP was assessed in the BFA-induced aggregates. After a 30 min BFA treatment (50 μM), a co-accumulation of PRP3-GFP and the endocytic tracer FM4-64, an endocytic tracer, was observed in BFA compartments (**Figures 2A–C**). To discriminate between PRP3-GFP signals derived from the secretory pathway and those from an endocytic pathway, PRP3 biosynthesis was inhibited using cycloheximide (CHX). Seedlings were incubated for 90 min with CHX (50 μM), then for 60 min with BFA (50 μM) and CHX (50 μM). PRP3-GFP was still present in BFA compartments after treatment (**Figures 2D–F**), indicating that PRP3-GFP is being actively endocytosed. Alternatively, seedlings pretreated with Tyrphostin A23 (TyrA23; 50 μM), a clathrin-mediated endocytosis inhibitor, and then co-treated with TyrA23 (50 μM) and BFA (50 μM) displayed greatly reduced PRP3-GFP signaling in BFA bodies (**Figures 2G–I**). As both PRP3-GFP and FM4-64 signals were reduced, it is probable that the protein is being endocytosed through a clathrin-dependent mechanism. Furthermore, the evidence indicates that SYP123 is involved in PRP3 polarization within the root hair tip cell wall and that both proteins are recycling via endocytosis.

**FIGURE 3:**
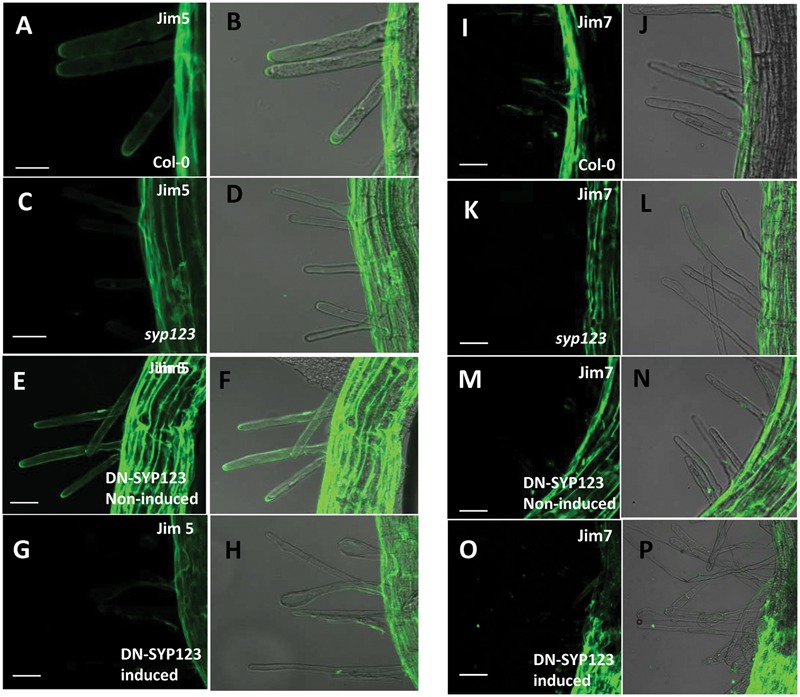
**SYP123 defective plants exhibit altered distribution of JIM5 epitopes.** Immunodetection in 5-days-old roots using the JIM5 antibody **(A–H)**, which detects de-esterified pectins, or JIM7 **(I–P)**, which detects esterified pectins. The Jim5 signal was strong in the root hair of Col-0 wild-type (WT) plants, and Jim7 signal was absent in the tips of *syp123* and in the DN (induced with dexamethasone and heat). Scale bars are indicated in the figure. Scale bar = 20 μm.

Immunolabeling experiments were also performed using a battery of different cell wall antibodies. The results revealed that several epitopes remained unchanged in the *syp123* mutant and induced in DN-SYP123 plants (Supplementary Figure S3). Alternatively, labeling with JIM5, a monoclonal antibody that detects homogalacturonan with a low degree of methyl esterification (Knox et al., 1990; Cavalier et al., 2008), indicated high concentrations at the tip of growing root hairs in the *Arabidopsis* ecotype Columbia (Col-0) and in non-induced DN-SYP123 plants as previously described (Cavalier et al., 2008). However, signaling was absent in the *syp123* mutant and in induced DN-SYP123 (**Figures 3A–H**). On the other hand, labeling with JIM7, an antibody specific for homogalacturonan with a high degree of methyl esterification (Knox et al., 1990; Cavalier et al., 2008), displayed labeling as previously described, in the base of Col-0 growing root hairs (Šamaj et al., 1999; **Figures 3I,J**). Nevertheless, the observed signal was similar between Col-0, the non-induced and induced DN-SYP123, and the *syp123* mutant plants (**Figures 3I–P**). Therefore, root hairs defective in SYP123 activity exhibit deficiency in both protein and cell wall polysaccharides composition.

### The *syp123* Mutant and DN-SYP123 Are More Susceptible to Plant Pathogens

Plants defective in SYP123 function grown in non-autoclaved soil showed increase sensitivity to occasional pathogens present in our growth chambers. Since the cell wall is a barrier limiting pathogen access to plant cells (Vacheron et al., 2013; Malinovsky et al., 2015) and since plasma membrane syntaxins have been related to plant defense responses this study hypothesized that a relationship exists between SYP123 function and plant immunity. To further characterize this connection, *syp123* mutant, DN-SYP123, and WT plants grown in non-sterile soil were challenged with pathogenic (*P. syringae* pv. *tomato* strain DC3000) and non-pathogenic bacteria (fluorescent *Pseudomonas* spp.).

**FIGURE 4:**
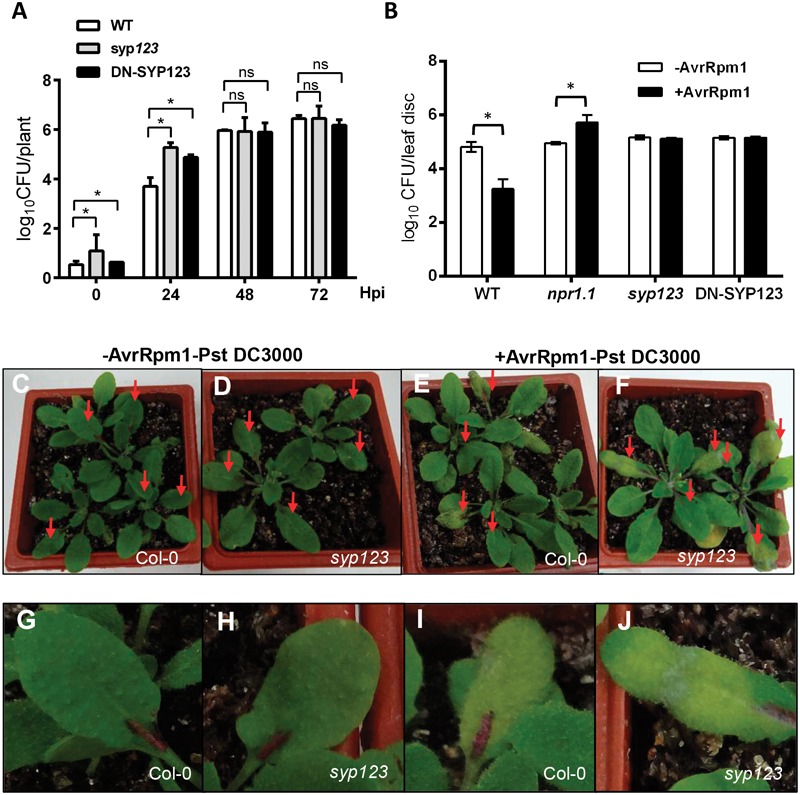
***syp123* mutants are susceptible to *Pseudomonas syringae* infection. (A)** Leaves of the indicated genotypes were infiltrated with *Pst* DC3000 and the number of colony forming units (CFUs) per leaf area determined after serial dilution and cultivation on selective media. Mean and standard deviation of triplicates at 0, 2, and 5 days post-infection (dpi) are shown. Different letters represent statistical differences during the progress of the infection (non-significant differences between genotypes were recorded with ANOVA and Tukey’s post-test). **(B)** Whole leaves of 4-weeks-old, soil-grown, WT Col-0 and mutant plants were infiltrated with *Pst* AvrRpm1 to trigger SAR (+AvrRpm1); a solution of 10 mM MgCl_2_ was used as mock (-AvrRpm1). After 24 h the systemic leaves were infiltrated with *Pst* DC3000. Bacterial growth was monitored 72-h post-infection. Error bars represent standard deviation from six samples. Different letters represent statistical differences between the genotypes and treatment at *p* < 0.01 (Tukey’s test). All experiments were performed in triplicate with similar results. **(C–F)** Pictures of WT and *syp123* mutant plants showing the phenotype 72-h post-infection. **(G–J)** A Close up of lesions in infected leaves.

The *syp123* mutant, DN-SYP123, and Col-0 WT plants were infected with the hemibiotrophic pathogen *P. syringae* pv. *tomato* strain DC3000 (*Pst* DC3000). First, plants were tested for basal defenses post-inoculation with a high dose (OD_600_ = 0.1) of virulent *Pst* DC3000. At 24-h post-inoculation *syp123* mutant and DN-SYP123 plants showed an increase bacterial growth (**Figure 4A**). Nevertheless, after 48 and 72 h post-inoculation *syp123* mutant, DN-SYP123, and WT plants exhibited similar bacterial growth. Indicating that the SYP123 defective plants are able to fight the infection over time. To test if plants lacking SYP123 could induce systemic acquired resistance (SAR), basal leaves from plants were infected with an avirulent strain of *Pst* DC3000 carrying the avrRpm1 gene, followed by injection with virulent *Pst* DC3000. The *syp123* mutant and DN-SYP123 plants were more susceptible to infection than the control WT plants (**Figure 4B**). The *npr1.1* mutant plants were used as a hypersensitive genotype (Cao et al., 1994). Increased susceptibility was detected through higher bacterial concentrations 72 h post-infection (**Figure 4B**), as well as through significantly more severe disease symptoms (**Figures 4C–J**). This suggested a deficiency in the systemic defense response but only diminished early basal defense resistance.

The connection between root hair and the SAR could be traced to a deficiency mounting the ISR in the roots as represents an additive mechanism (Yi et al., 2013). Interestingly, the *npr1.1* mutant plants previously shown to be unable of mounting both ISR or SAR responses (Iavicoli et al., 2003) exhibited increased susceptibility to *Pst* DC3000 compare to SYP123 deficient plants (**Figure 4B**). Root hair are important components of the PGPR-root response (Prieto et al., 2011; Mercado-Blanco and Prieto, 2012) although the specific mechanisms involved remain unclear. Interestingly, the *prp3* knock-out mutant (Larson et al., 2014), another root hair specific protein, displays grater sensibility to *Pst* DC3000 avrRpt2 challenge (Supplementary Figure S4).

**FIGURE 5:**
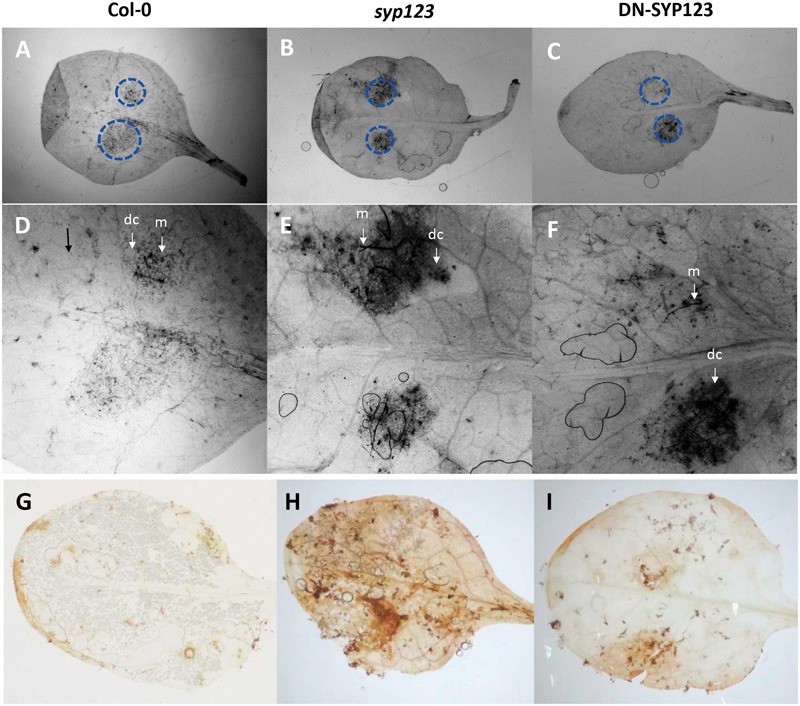
***Botrytis* infection assays in seedlings with a defective SYP123 function. (A–F)** The leaves from Col-0, *syp123* mutant, and DN-SYP123 plants were inoculated with a spore suspension of *Botrytis cinerea*. Leaves were stained with trypan blue at 4 days post inoculation (dpi). Dotted circles indicate the borders of spore inoculation sites in control Col-0 **(A,D)**, *syp123* mutant **(B,E)**, and DN-SYP123 **(C,F)**. Stained plant cells surrounding the fungal mycelium (m) indicates dead plant cells (dc). **(D–F)** Shows a higher magnification of the inset from the superior panel at 4 dpi. **(G–I)** Production of H_2_O_2_ is visualized by DAB staining in *Botrytis* inoculated leaves at 4 dpi. The brown precipitate shows DAB polymerization at the site of H_2_O_2_ production. Representative leaves detached from inoculated Col-0 **(A)**, *syp123*, and DN-SYP123 plants are shown. All the experiments were performed in triplicate with similar results.

Systemic acquired resistance and ISR differ in their effectiveness against different types of attackers. For example, only ISR is active against the *B. cinerea* (Ton et al., 2002) and *P. infestans* (Yan et al., 2002). Leaves inoculated with the necrotrophic fungal pathogen *B. cinerea* (Chilean isolate) showed patches of cell death or necrosis surrounding infection sites with an increase in cell death in both *syp123* mutant and DN-SYP123 plants at 4 dpi compared with WT. Staining of infected WT leaves showed that the fungal mycelium was restricted to areas surrounded by a ring of dead cells (**Figures 5A,D**) accordingly with previous reports (Rowe et al., 2010). Leafs of *syp123* mutant and DN-SYP123 showed an increase in mycelium development compared to WT plants (**Figures 5B–F**). This reaction to the infection was accompanied by the induction of reactive oxygen species (ROS) at 4 dpi as is revealed by an increase in the levels of H_2_O_2_ evidenced by 3,3′-diaminobenzidine staining (**Figures 5G–I**). The amount of cell death was closely related to the extent of DAB staining indicating that SYP123 deficient plants are more susceptible to *Botrytis* infection.

**FIGURE 6:**
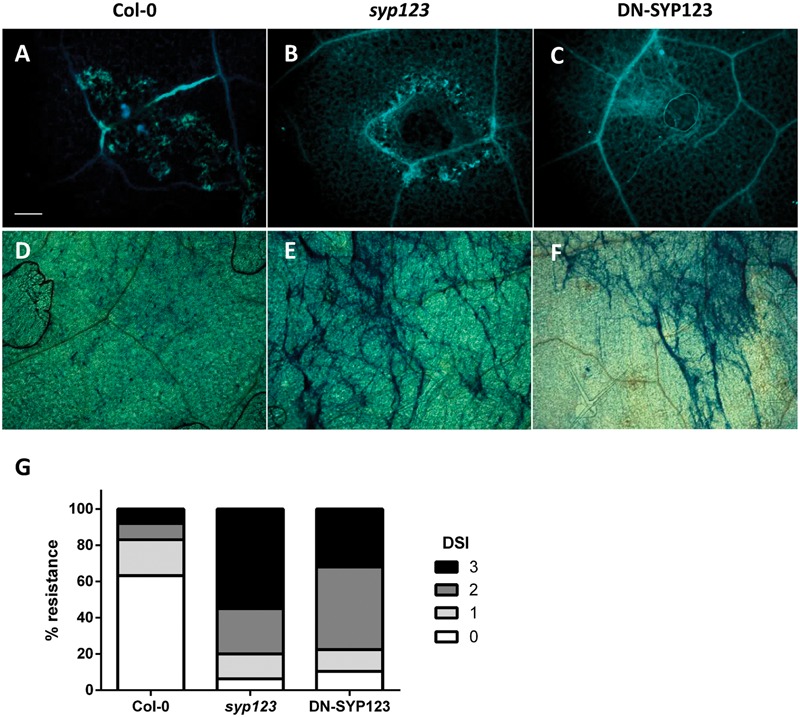
***Phytophthora* infection assays in seedlings with a defective SYP123 function. (A–F)** Response to *Phytophthora infestans* inoculation. Callose staining with aniline blue in *Arabidopsis* WT Col-0 **(A)**, *syp123* mutant **(B)**, and DN-SYP123 **(C)** 7 dpi. Callose accumulated at the site of inoculation in all plants. A small amount of callose was deposited in Col-0 **(A)**, while widespread callose deposition occurred in *syp123* **(B)** and DN-SYP123 **(C)** leaves. Trypan blue staining of the inoculated leaves of Col-0 **(D)**, *syp123* **(E)**, and DN-SYP123 **(F)** at 7 dpi. Scale Bar = 50 μm. **(G)** Disease severity index (DSI) presented by numbers from “0” to “3”, as follows: 0 = no disease symptoms; 1 = a few spots observed within the droplet/plug zone; 2 = confined lesion covering droplet/plug zone; 3 = outgrowing lesion. Mean disease resistance scores were transformed into percentage values for comparison of replicate inoculations.

*Phytophthora infestans* penetrated epidermal cells displaying typical features including granulated cell cytoplasm, thickened cell walls as the aniline blue stain reveals (**Figure 6A**; Huitema et al., 2003). All responses are typically limited to the penetrated epidermal cell. However, *syp123* mutant and DN exhibited macroscopic cell death with lesions 7 dpi and extended callose deposition (**Figures 6B,C**). A few stained trypan blue epidermal cells appeared in WT plants (7 dpi; **Figure 6D**). In contrast, the trypan blue dye was precipitated in *syp123* mutant and DN epidermal and mesophyll cells where the zoospore droplets had been applied on the leaves (**Figures 6E,F**). Thus, while little penetration of *P. infestans* hyphae into epidermal cells was observed in the WT plant, more penetration and growth of *P. infestans* in the *syp123* mutant and DN-SYP123 plants were observed (**Figure 6G**). Therefore, it is possible that the root hair t-SNARE SYP123 is involved in the priming of ISR response. These data indicate a connection between root hair cell elongation and cell wall composition in the whole plant response to pathogens. Nevertheless, the specific mechanisms remain unclear, and the observed phenotypes remain to be detailed in a future study.

### The *syp123* Deficient Plants Exhibited Altered Induced Systemic Resistance

Induced systemic response-positive plants react faster and more strongly to pathogen attack by inducing defense genes as *PR1, PDF1.2, MYB72*, and *MYC2* (De Meyer and Hofte, 1997; Yan et al., 2002; Ryu et al., 2003). Thus, ISR priming in response to PGPR was evaluated in SYP123 deficient plants through assessing the gene expressions of *PR1, PDF1.2, MYB72*, and *MYC2*.

**FIGURE 7:**
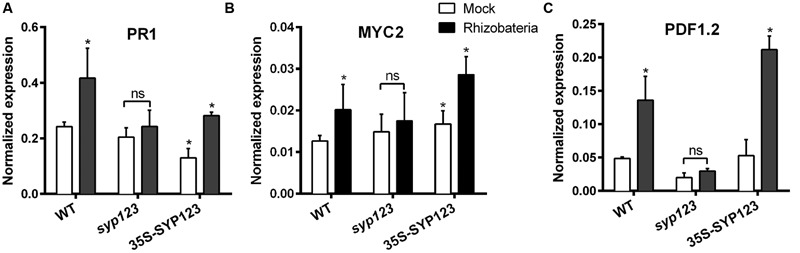
**Induced systemic response (ISR)-marker genes expression is affected by SYP123 levels.** The mRNA levels of **(A)** *PR1* **(B)** *MYC2*, and **(C)** *PDF1.2* ISR marker genes were measured in the rosette leaves of WT Col-0, *syp123*, or *syp123*x35S:SYP123 plants. The plants were grown for 7 days and then the roots were inoculated with beneficial rhizobacteria *Pseudomonas* spp. At 8 dpi the leaves were collected and the expression of ISR marker genes was analyzed using qRT-PCR. Asterisks indicate statistically significant differences (Student’s *t*-test, *p* < 0.01) compared to the control.

*Pseudomonas* spp. fluorescent can be isolated from the rhizosphere of many plant species (Keel et al., 1996; McSpadden Gardener et al., 2005; Weller et al., 2007). ISR priming by *Pseudomonas* spp. isolated-PGPR was investigated in the Col-0, *syp123* mutant, and 35S-SYP123 over-expressor plants (*syp123* background). Seven-days-old seedlings grown vertically in an agar-solidified medium were inoculated with *Pseudomonas* spp.-isolated PGPR suspensions applied 5 cm from the root tip. After 7 days of co-cultivation, qRT-PCR was used to analyze the expression patterns of *PR1, MYC2*, and *PDF1.2* in *Arabidopsis* Col-0, *syp123* mutant, and 35S-SYP123 plants. The relative expression of the salicylic acid inducible gene *PR1* in Col-0 leaves was significantly higher in rhizobacteria-treated plants, which supported previous data on salicylic acid involvement in ISR (**Figure 7**). Similarly, the jasmonic acid and ethylene inducible marker gene *PDF1.2* and the jasmonic acid -inducible gene *MYC2* showed significantly higher relative expressions in treated Col-0 plants compared to mock controls (**Figure 7**). Moreover, 35S-SYP123 over-expressor plants exhibited increased levels of *PR1, PDF1.2*, and *MYC2* mRNA compared to control plants. In contrast, transcript levels of these genes were much lower in rhizobacteria-treated *syp123* mutant plants. The decreased expression in the *syp123* mutant indicates a reduced ISR priming.

These results point an important role for SYP123 function in the priming of ISR defense upon colonization by PGPR.

## Discussion

### SYP123 Affects the Arrangement of Proteins and Polysaccharides during Root Hair Tip Growth

SYP123 is specifically expressed in the root hair cells and polarized accumulated at the plasma membrane of the tip of growing root hairs (Enami et al., 2009). Root hairs elongate by tip growth by remodeling the preexistent cell wall to allow the active incorporation of new materials, recovering excess plasma membrane and recycling proteins. Interestingly, root hairs of *syp123* mutant plants are shorter pointing a role for this protein in tip growth related processes (Ichikawa et al., 2014). Consistently, we founded that the structural cell wall protein PRP3 is no longer detectable at the surface of tip growing root hairs in *syp123* mutant and the DN-SYP123. Additionally, JIM5 polysaccharides epitopes, homogalacturonans with a low degree of methyl esterification, also presented altered distribution at the root hair tips of these plants. JIM5-reactive pectins are localized at the innermost part of cell walls adjacent to the plasma membrane and can be internalized into BFA compartments in root epidermal cells (Baluška et al., 2002; Paciorek et al., 2005). In contrast, JIM7-reactive pectins do not show this endocytic localization (Baluška et al., 2002). As SYP123 it is also recycling in BFA-sensitive endosomes (Ichikawa et al., 2014) the observed mutant phenotypes could be related to a role in the polarization of cell wall material at the root tip. Accordingly, PRP3 can also be found in BFA compartments and is endocytosed by a clathrin dependent mechanism.

This is the first link between a plasma membrane t-SNARE and cell wall deposition and recycling. Further studies are necessary to dissect the mechanism involving SYP123 action that could lead to additional discoveries in the field of protein polarization and cell wall assembly.

### SYP123 Is Involved in the Plant Immunity Response

Root hairs and lateral roots play important roles in the colonization by PGPR (Pothier et al., 2007; Galland et al., 2012; Venus and Oelmüller, 2013). Several colonizing rhizobacterial strains have been shown to induce systemic resistance (ISR). ISR prime induces expression of defense genes as *PR1, PDF1.2, MYB72*, and *MYC2* (De Meyer and Hofte, 1997; Yan et al., 2002; Ryu et al., 2003). SYP123 overexpressor plants showed increased expression of ISR priming genes compared with WT plants in response to rhizobacteria *Pseudomonas* spp. On the contrary, *syp123* mutant plants exhibit a decreased expression of the ISR priming marker genes indicating a direct correlation between SYP123 function and ISR priming of defense.

The induced resistance trigger in the roots is manifested in the whole plant as a reduction in disease severity upon subsequent infection by a pathogen. The spectrum of diseases to which PGPR-elicited ISR confers enhanced resistance overlaps partly with that of pathogen-induced SAR. Accordingly, *syp123* mutant and DN-SYP123 plants were unable to mount an effective SAR, compared to Col-0 WT plants against the bacterial pathogen *P. syringae* pv. *tomato* strain DC3000. Even do both, ISR and SAR, represent a state of enhanced resistance of the plant pathogens are differentially sensitive to the resistances activated by each of these signaling pathways. SYP123 defective plants are more susceptible to the infection by the necrotrophic fungal pathogen *B. cinerea*, and the non-host pathogen *P. infestans.* Accordingly, ISR but no SAR prime active defenses against these two pathogens (Ton et al., 2002; Yan et al., 2002).

All together these data indicate a direct relationship between SYP123 function and ISR priming of defense.

### Root Hair Cell Wall and PGPR Response

The connection between root hair and the systemic defense could also be related to a deficient ISR in the roots. Certain fluorescent *Pseudomonas* isolates promote plant growth by producing plant growth-promoting substances (i.e., PGPR), and PGPR has been found to beneficially affect plant health. Indeed, these rhizobacteria can induce resistance against fungal, bacterial, and viral diseases and insect pests (Maurhofer et al., 1998; Chen et al., 2000; Pieterse et al., 2014).

Root hair cell wall is a prime interactor with soil microbiota (Vacheron et al., 2013). PGPR induce an increase in root hair number and growth forming a biofilm in addition to ISR priming (Rudrappa et al., 2010). During these processes, several genes encoding for cell wall modifying-enzymes, such as pectin methyl esterase, are up-regulated, thereby contributing to reduced cell wall rigidity (Zhang et al., 2007; Bellincampi et al., 2014). Due to a lack of homogalacturonan with low methyl esterification in the root hair tips of SYP123 defective plants, the differential rigidity of the cell wall could interfere with rhizobacteria interactions. Additionally, in response to pathogen infection, proline-rich proteins are rapidly insolubilized, strengthening the cell wall (Brisson et al., 1994). Therefore, the altered PRP3 localization in SYP123 defective plants could affect the onset of ISR. Interestingly, the *prp3* knockout mutant (Larson et al., 2014) displayed greater susceptibility to an avirulent strain of *P. syringae*. Since PRP3 is exclusively expressed in root hair cells, the increased susceptibility of the mutant supports our hypothesis that the correct localization of PRP3 at the cell wall is involved in the onset of ISR.

Considering that some host responses are necessarily mediated by receptor molecules associated with or embedded in the plasma membrane, SYP123 has another potential role in the onset of ISR. The localization or internalization of these receptors could be affected in SYP123 defective plants, which would impair the ISR response. Overall, the data in this work strongly suggest that the root hair specific t-SNARE SYP123 function is important for the onset of ISR.

## Conclusion

The results indicated that root hairs are central to rhizobacteria priming of an ISR pathway of *Arabidopsis* plants. Our data support that SYP123 functions in the plasma membrane of growing root hairs are important for the correct localization and distribution of proteins and polysaccharides in the cell wall. This study is the first to indicate that a root hair-specific protein related to cell wall protein and polysaccharide distribution is involved in the onset of a rhizobacteria-promoted induced system response. Therefore, t-SNAREs are implicated in both pathogenic and beneficial plant/microbe interactions. These are important findings linking primary perception at the root hairs with the downstream activation of plant defense responses. Future studies will focus on describing the specific mechanisms involved in these processes.

## Author Contributions

CR-F and FB-H, conceived and designed the experiments. CR-F, HS-G, OS, CR, PA-S, and SS-A performed the experiments. CR-F and FB-H analyzed the data. CR-F, AO, and FB-H wrote the manuscript.

## Conflict of Interest Statement

The authors declare that the research was conducted in the absence of any commercial or financial relationships that could be construed as a potential conflict of interest.

## Footnotes

1 http://rsb.info.nih.gov/ij/
