# Supplementary material for: The Root Hair Specific SYP123 Regulates the Localization of Cell Wall Components and Contributes to Rizhobacterial Priming of Induced Systemic Resistance
Source: Front Plant Sci. 2016 Jul 26;7:1081. doi: 10.3389/fpls.2016.01081 (PMC4961009; doi:10.3389/fpls.2016.01081)
Supplement: Supplementary file 1 [file Presentation_1.PPTX]

## Slide 1
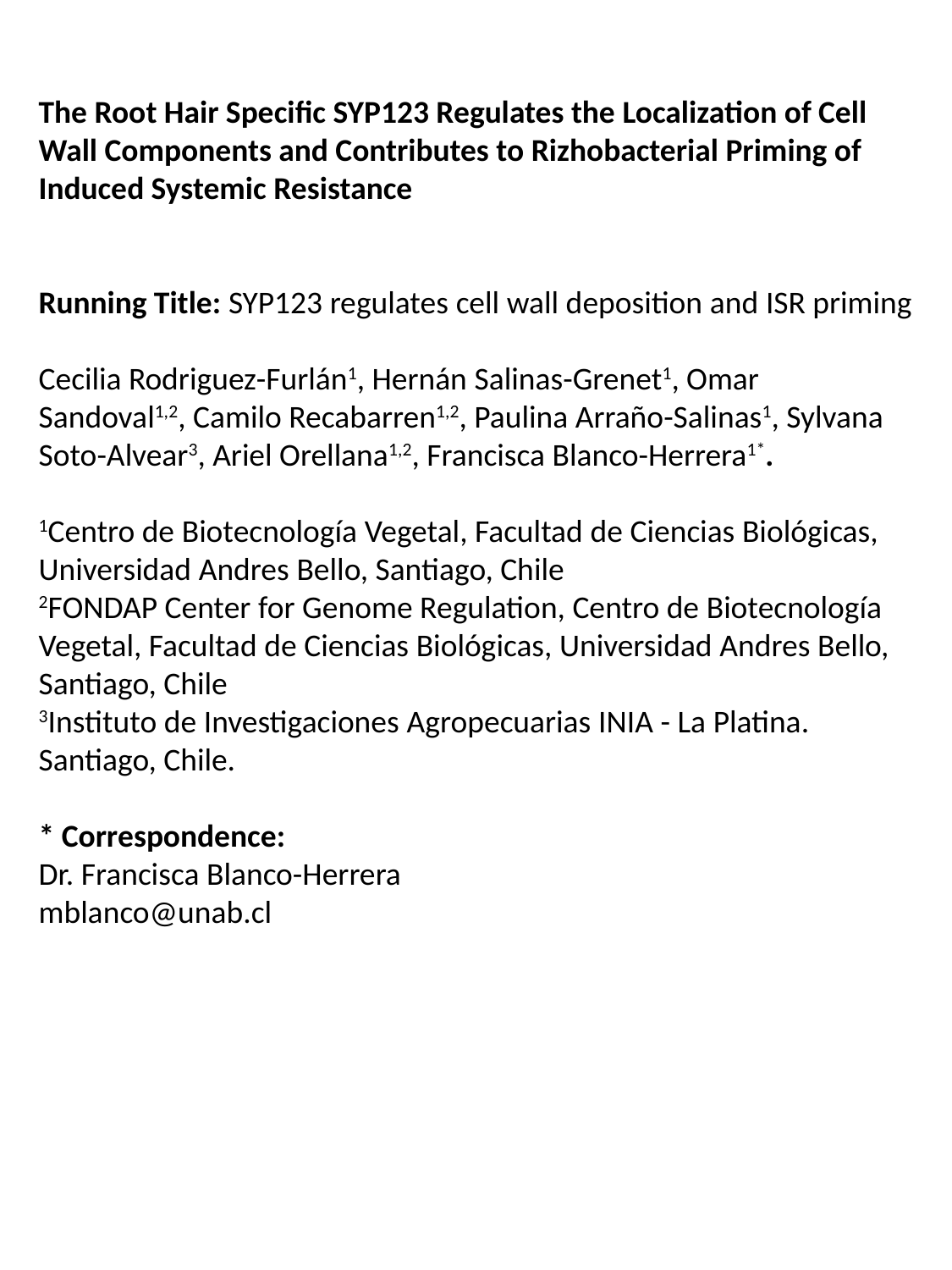

The Root Hair Specific SYP123 Regulates the Localization of Cell Wall Components and Contributes to Rizhobacterial Priming of Induced Systemic Resistance
Running Title: SYP123 regulates cell wall deposition and ISR priming
Cecilia Rodriguez-Furlán1, Hernán Salinas-Grenet1, Omar Sandoval1,2, Camilo Recabarren1,2, Paulina Arraño-Salinas1, Sylvana Soto-Alvear3, Ariel Orellana1,2, Francisca Blanco-Herrera1*.
1Centro de Biotecnología Vegetal, Facultad de Ciencias Biológicas, Universidad Andres Bello, Santiago, Chile
2FONDAP Center for Genome Regulation, Centro de Biotecnología Vegetal, Facultad de Ciencias Biológicas, Universidad Andres Bello, Santiago, Chile
3Instituto de Investigaciones Agropecuarias INIA - La Platina. Santiago, Chile.
* Correspondence:
Dr. Francisca Blanco-Herrera
mblanco@unab.cl

## Slide 2
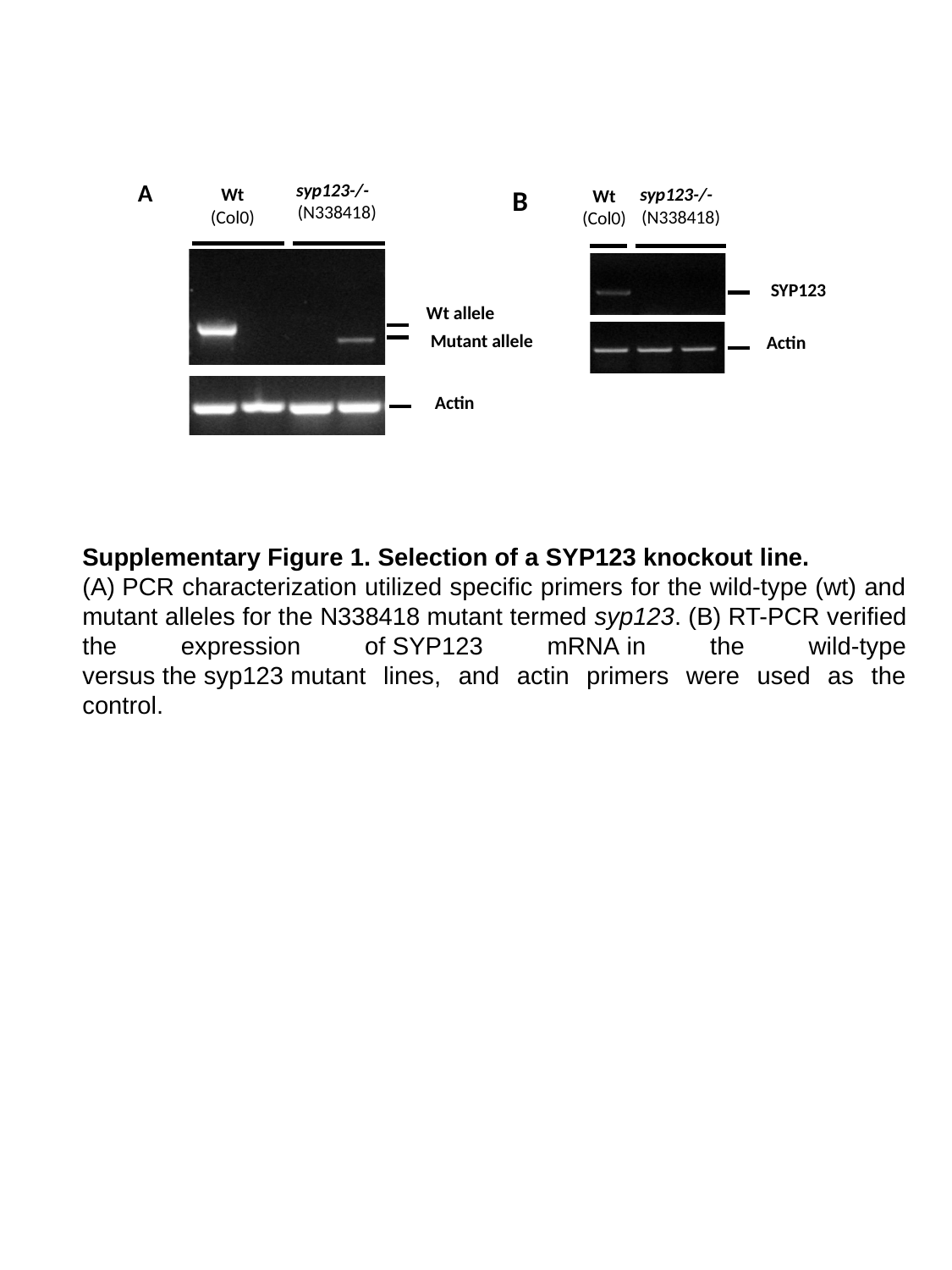

A
syp123-/- (N338418)
Wt
(Col0)
B
syp123-/- (N338418)
Wt
(Col0)
SYP123
Wt allele
Mutant allele
Actin
Actin
Supplementary Figure 1. Selection of a SYP123 knockout line.
(A) PCR characterization utilized specific primers for the wild-type (wt) and mutant alleles for the N338418 mutant termed syp123. (B) RT-PCR verified the expression of SYP123 mRNA in the wild-type versus the syp123 mutant lines, and actin primers were used as the control.

## Slide 3
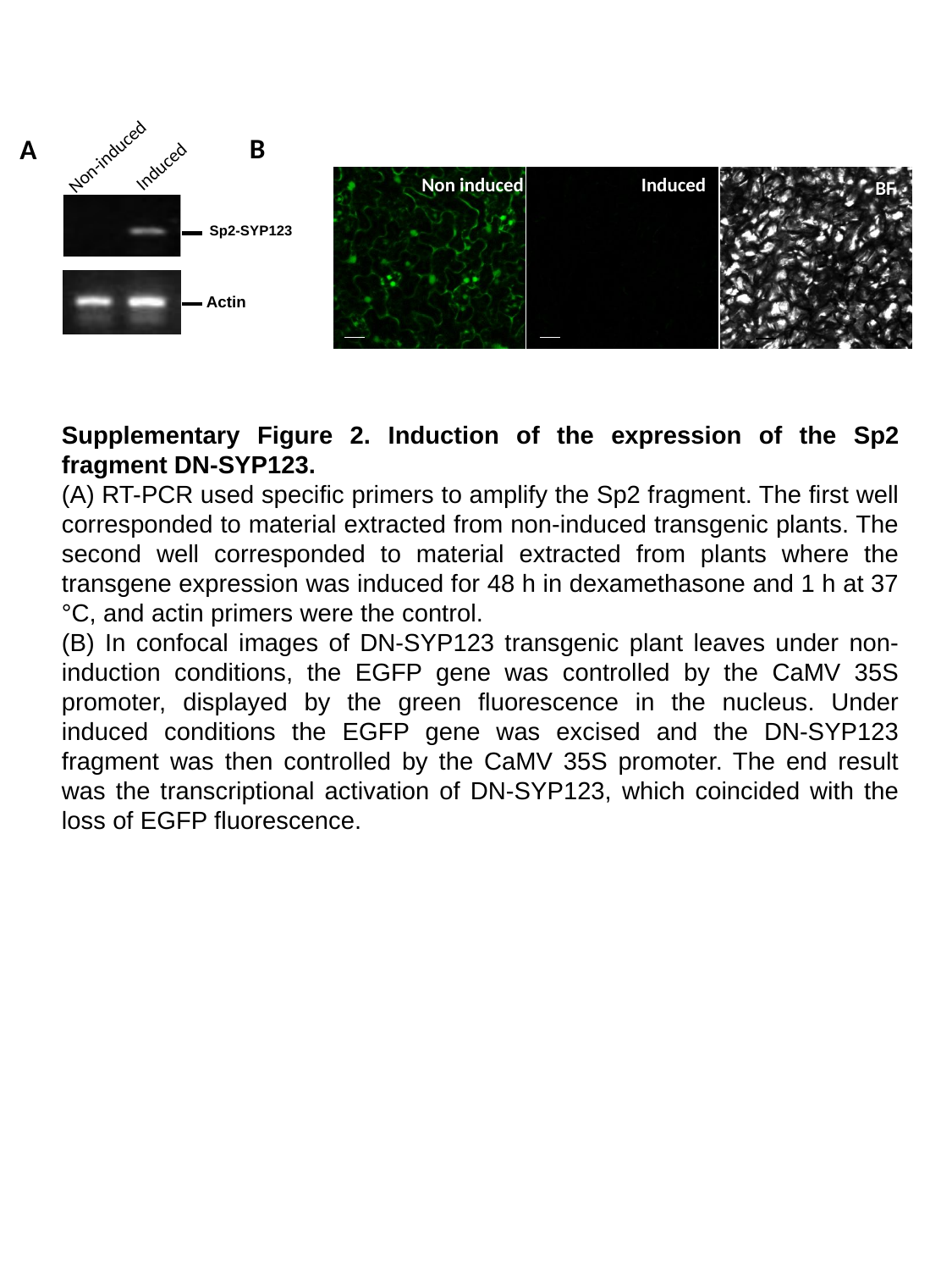

B
A
Non-induced
Induced
Non induced
Induced
BF
Sp2-SYP123
Actin
Supplementary Figure 2. Induction of the expression of the Sp2 fragment DN-SYP123.
(A) RT-PCR used specific primers to amplify the Sp2 fragment. The first well corresponded to material extracted from non-induced transgenic plants. The second well corresponded to material extracted from plants where the transgene expression was induced for 48 h in dexamethasone and 1 h at 37 °C, and actin primers were the control.
(B) In confocal images of DN-SYP123 transgenic plant leaves under non-induction conditions, the EGFP gene was controlled by the CaMV 35S promoter, displayed by the green fluorescence in the nucleus. Under induced conditions the EGFP gene was excised and the DN-SYP123 fragment was then controlled by the CaMV 35S promoter. The end result was the transcriptional activation of DN-SYP123, which coincided with the loss of EGFP fluorescence.

## Slide 4
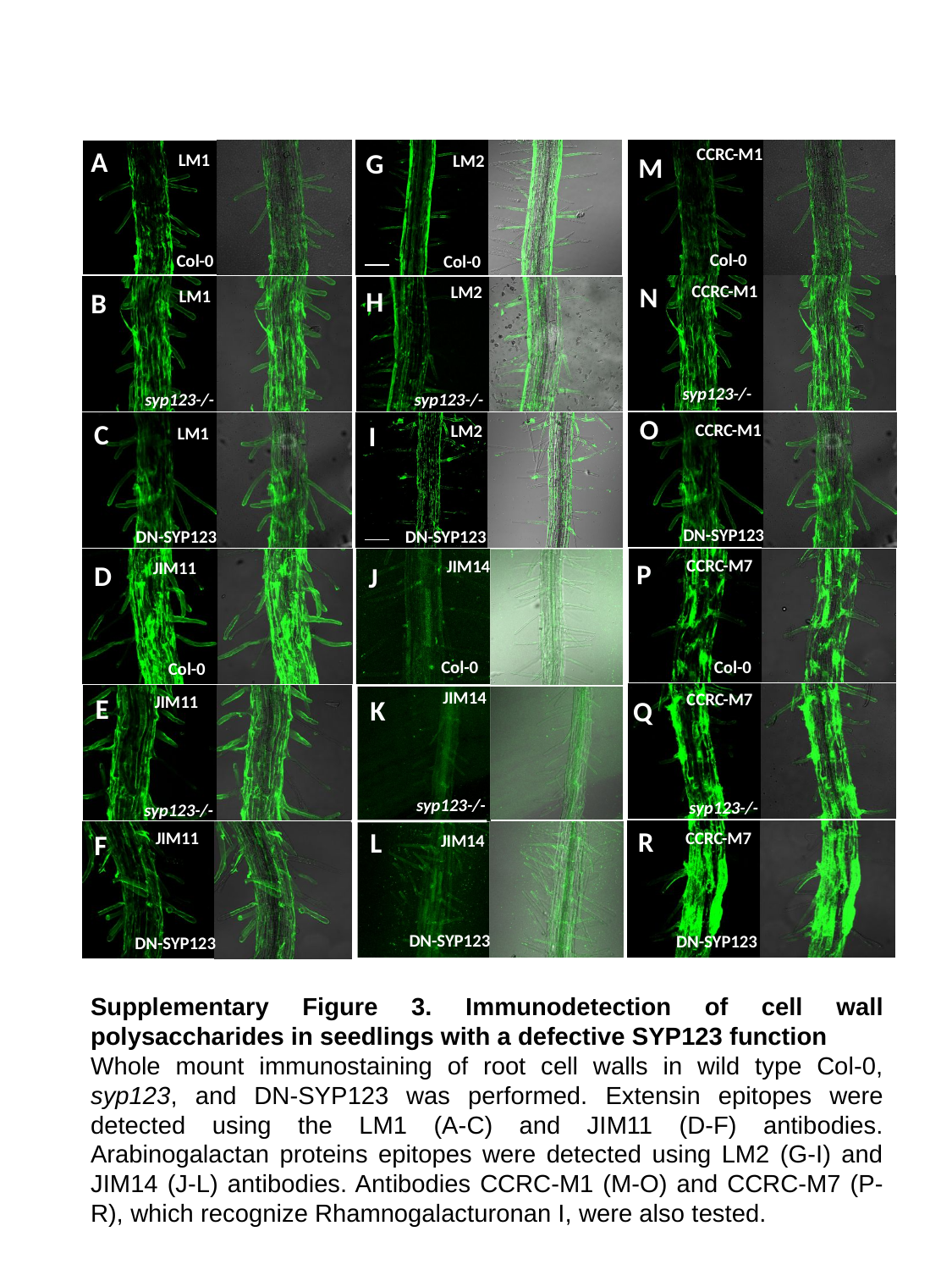

CCRC-M1
A
G
LM1
Col-0
LM1
syp123-/-
LM1
DN-SYP123
LM2
M
Col-0
Col-0
N
CCRC-M1
LM2
LM2
H
B
syp123-/-
syp123-/-
O
C
I
CCRC-M1
LM2
DN-SYP123
DN-SYP123
CCRC-M7
JIM14
P
JIM11
D
J
Col-0
Col-0
Col-0
JIM14
CCRC-M7
JIM11
E
K
Q
syp123-/-
syp123-/-
syp123-/-
R
L
CCRC-M7
F
JIM11
JIM14
DN-SYP123
DN-SYP123
DN-SYP123
Supplementary Figure 3. Immunodetection of cell wall polysaccharides in seedlings with a defective SYP123 function
Whole mount immunostaining of root cell walls in wild type Col-0, syp123, and DN-SYP123 was performed. Extensin epitopes were detected using the LM1 (A-C) and JIM11 (D-F) antibodies. Arabinogalactan proteins epitopes were detected using LM2 (G-I) and JIM14 (J-L) antibodies. Antibodies CCRC-M1 (M-O) and CCRC-M7 (P-R), which recognize Rhamnogalacturonan I, were also tested.

## Slide 5
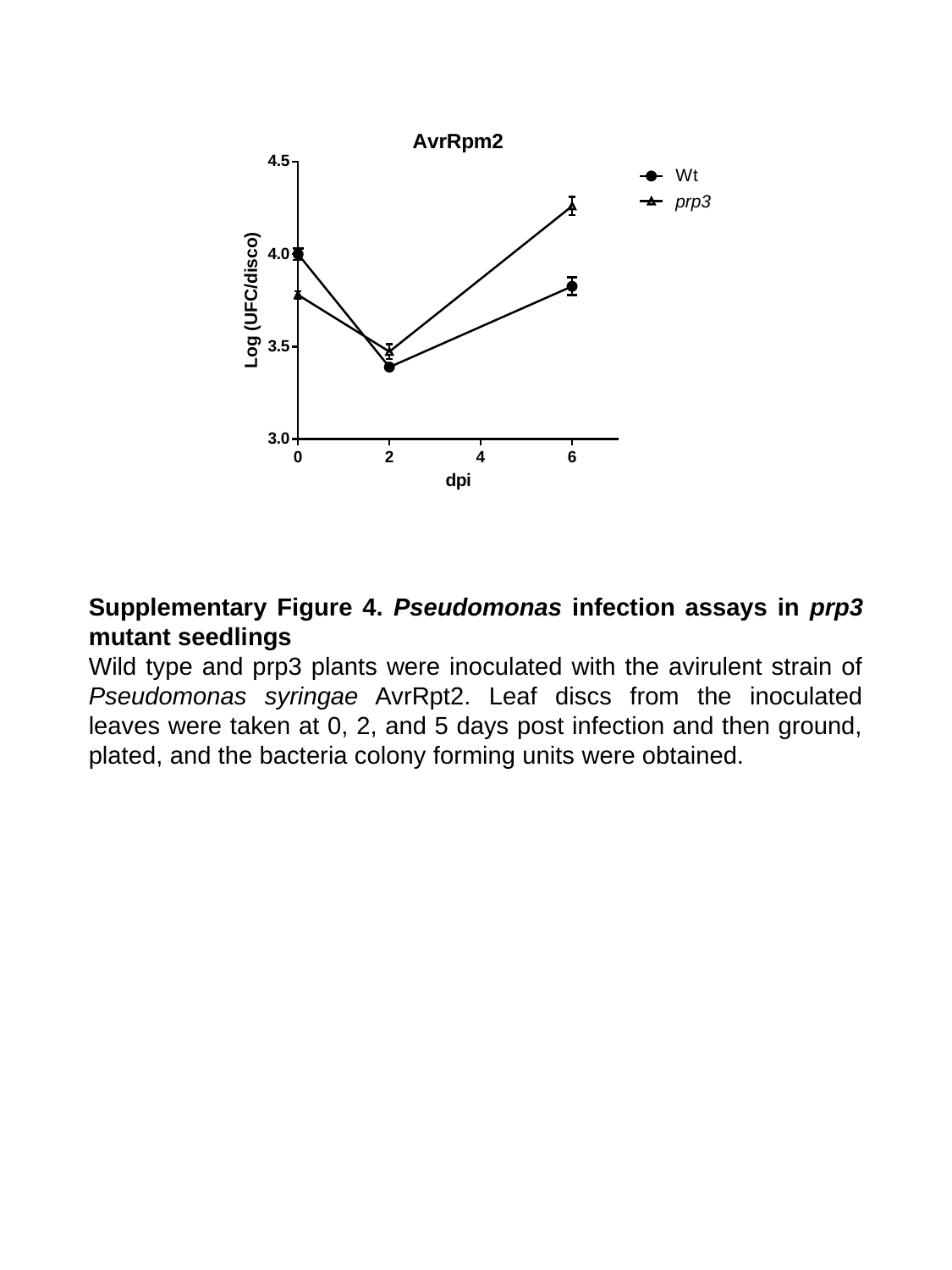

Supplementary Figure 4. Pseudomonas infection assays in prp3 mutant seedlings
Wild type and prp3 plants were inoculated with the avirulent strain of Pseudomonas syringae AvrRpt2. Leaf discs from the inoculated leaves were taken at 0, 2, and 5 days post infection and then ground, plated, and the bacteria colony forming units were obtained.
